# Conditions for interprofessional education for students in primary healthcare: a qualitative study

**DOI:** 10.1186/s12909-018-1245-8

**Published:** 2018-06-04

**Authors:** Carrie Tran, Päivi Kaila, Helena Salminen

**Affiliations:** 1 Department of Neurobiology, Care Sciences and Society, Division of Family Medicine and Primary Care, Karolinska Institutet, Alfred Nobels Allé 23, 141 83 Huddinge, Sweden; 2 Department of Neurobiology, Care Sciences and Society, Division of Nursing, Karolinska Institutet, Alfred Nobels Allé 23, 141 83 Huddinge, Sweden

**Keywords:** Interprofessional education, Primary healthcare, Healthcare students, Qualitative study, Content analysis

## Abstract

**Background:**

Primary healthcare in Sweden and worldwide has a diverse structure with many kinds of healthcare units involved. This is a challenge for collaboration between different professions in primary healthcare, as the different healthcare professions often work in silos. Interprofessional education (IPE) in the context of primary healthcare is less studied than IPE at hospitals and most of the studies in primary healthcare have focused on collaboration between general practitioners and nurses. The aim of this study was to describe how healthcare students perceived conditions for IPE in primary healthcare.

**Methods:**

Qualitative group interviews were used and a total of 26 students, recruited on a voluntary basis participated in four group interviews with students mixed from study programmes in nursing, physiotherapy, occupational therapy and medicine. Students from the study programme in medicine were in their second to eleventh semesters of 11 semesters in total, whilst students from the occupational therapist, physiotherapist and nursing programmes were in their fourth to sixth of six semesters in total.

**Results:**

Our findings indicated one theme: Students perceived a need for support and awareness of IPE from both study programmes and clinical placements*.* Five categories were found to belong to the theme. *Students’ tunnel-vision focus on their own profession* may have affected their ability to collaborate with students from other professions. The nature of the *patients’ healthcare problems* decided if they were perceived as suitable for IPE. C*linical supervisors’ support for and attitude towards IPE* were important. The *hierarchy between different professions was* perceived as a hindrance for seeking help from the other professions. The students asked for more *collaboration between different study programmes,* in order to gain knowledge about the roles and responsibilities of the other professions.

**Conclusions:**

In conclusion, students in this study considered it essential for different study programmes and clinical placements to be more aware of the opportunities for and importance of IPE. The study identified conditions that were required for IPE in primary healthcare that may be helpful for healthcare teachers and clinical supervisors to better understand how students perceive IPE in primary healthcare, thus facilitating the planning of IPE.

**Electronic supplementary material:**

The online version of this article (10.1186/s12909-018-1245-8) contains supplementary material, which is available to authorized users.

## Background

Patient safety and quality of care would be improved if there was increased collaboration between healthcare professions [1, 2]. Most healthcare educations involve a large number of students and take place in isolation from each other, reducing possibilities for interaction between the different student categories [1, 3]. This tendency for both healthcare professions and educations to work in silos makes collaboration between different professions a challenge [3].

In Swedish primary healthcare, some healthcare professions rarely have direct contact via meetings in person about the individual patient’s care, since they do not work close enough to each other. Furthermore, most of the time students do not meet students from other professions since they do not have their clinical placements at the same time. IPE occurs by definition *“when students from two or more professions learn about, from and with each other to enable effective collaboration and improve health outcome”*. The World Health Organization stated that once the students understand how to work interprofessionally, they will have increased readiness to work collaboratively when entering a workplace [4].

According to the Declaration of Alma-Ata [5], primary healthcare is the first level of healthcare contact for individuals who live in the community. It brings healthcare as close as possible to where people live and work. The main health fields that primary healthcare deals with are preventive, curative, supportive and rehabilitative services. There were about 200 primary healthcare centres and a total of around 500 units (including rehabilitation, child and maternity care units) in primary healthcare in Stockholm, Sweden, when the present study was conducted. Most of the nurses and medical doctors work at primary healthcare centres, whilst occupational therapists and physiotherapists generally work at rehabilitation units. It is uncommon for primary healthcare centres and rehabilitation units to be co-located. Counsellors, psychologists and dietitians also work at some of the primary healthcare centres.

There has been an increased focus on IPE because there exists a growing recognition and evidence of communication and collaboration improvements in interprofessional teams [6]. Social engagement is important for the students’ learning process and ability to make sense of new information and ideas. Learning takes place in a community of practice [7] and is a part of a social practice. Through social interaction with peers and clinicians in a clinical environment, the students construct new knowledge and are introduced to their future profession by doing and acting.

There are several previous studies about IPE in primary healthcare [8] but there still exists a lack of research that relates to IPE involving several professions’ students similar to the present study. Kent & Keating [8] could only find two such studies in their systematic literature review from 2015. Moreover, according to San Martín-Rodríguez et al. [9], seven of ten studies have been conducted in hospital contexts but only three in primary healthcare settings. Collaboration in hospitals is usually easier since most of the staff work in the same building. Hammick, Freeth, Koppel, Reeves and Barr [10] reviewed more than 21 scientific papers, most of them in a hospital context. In Sweden, IPE had been studied in clinical education wards in hospitals [11–14]. It was difficult to find studies about IPE in primary healthcare in a Swedish context. We have only found one study in Sweden that investigated undergraduate students’ experiences of a full day practicing teamwork with a fictitious home care patient [15]. Most of the previous studies in primary healthcare have focused on creating interprofessional learning activities. Students have rarely taken part in creating IPE and their views of what is required for IPE to take place having not been previously explored in the context of primary healthcare. This indicates a gap in knowledge regarding how students perceive prerequisites for IPE in primary healthcare. This study used an interprofessional approach in methodology and had students in mixed groups from four different study programmes. The purpose of the present study was to describe how students from study programmes in nursing, occupational therapy, physiotherapy and medicine perceived the conditions for IPE in primary healthcare.

## Methods

### Design

This was a qualitative study and data was collected through group interviews. The data was analysed using qualitative content analysis since this method provided knowledge and gave new insight into how the students perceived conditions for IPE in primary healthcare [16]. In order to obtain rich data from the interactions between the students from the different study programmes, group interviews was chosen as a method. The analysis had an inductive approach and focused mainly on the manifest content.

### Context and participants

The context of this study was primary healthcare in Stockholm, Sweden. Nursing, physiotherapy, occupational therapy and medicine students from one medical university (Karolinska Institutet) had clinical placements in primary healthcare. Some nursing students were from two other universities in Stockholm that also had placements in primary healthcare. The period for these placements in primary healthcare varied between 1 and 6 weeks and took place at different periods of the semester. The students were recruited on a voluntary basis via information given to all four programmes, and the students passed on information to other students via their Facebook pages.

Four group interviews with five to nine students in each group were carried out as follows: two group interviews were conducted at the Centre of Family Medicine and the remaining two groups at one Academic Health Centre. Medicine students were in the second to eleventh semesters of their 11 semester study programme, whilst students from the occupational therapist, physiotherapist and nursing programmes were in their fourth to sixth of six semesters in total. The groups were mixed with the aim to get participants from different study programmes in the same interview. As all the participants still had the same student status, we did not anticipate any problems with hierarchy among the students. Three groups had students from all four study programmes included and one group had students from three study programmes (with no nursing students). The intention was only to include undergraduate students with prior experience of a clinical placement in primary healthcare but unintentionally three students who were included in the study had not had their clinical placements in primary healthcare. The majority had experience of IPE from their clinical placements at hospitals in interprofessional student wards.

### Data collection

A semi-structured interview guide was used consisting of five open-ended questions (Additional file 1): What does IPE mean to you? What possibilities do you see to learn with, from and about each other in primary healthcare? What hindrances do you see to learn from each other in primary healthcare? What can you learn from each other in primary healthcare? How can you learn with and from each other in primary healthcare? Two people with different roles conducted all four interviews. KB acted as a moderator while the third author (HS) acted as an observer. The interviews were recorded and transcribed verbatim. Each session lasted approximately 90 min. After the fourth group interview, the recorded data showed little new information and the data had little variation so saturation was considered to have been achieved. The data was collected in autumn 2012.

### Data analysis

Data was analysed using qualitative content analysis inspired by Krippendorff [16]. As there was not enough knowledge about how students perceived prerequisites for IPE in primary healthcare, an inductive approach was used. The analysis started by reading the text several times in order to get a sense of a whole. Sentences and phrases with content relevant to the aim known as “meaning units”, were highlighted. The analysis was first done by the first author (CT) identifying the meaning units from the text. CT then condensed the meaning units and gave names with codes and then sorted the data into categories [17]. The codes were aimed to stay as close as possible to the text and were then grouped into categories and higher order categories. All authors agreed that the main theme covered the categories. Graneheim & Lundman [17] described category as a group of content that shares commonality and a theme can be found in several categories, describing the underlying meanings. In order to increase trustworthiness, all authors who were from different professions identified main categories independently and then discussed similarities and differences until consensus was reached.

### Ethical considerations

The Ethical Review Board in Stockholm had no objections to the study. All participants were informed about the aim and method of the study and gave signed informed consent.

## Results

**Fig. 1:**
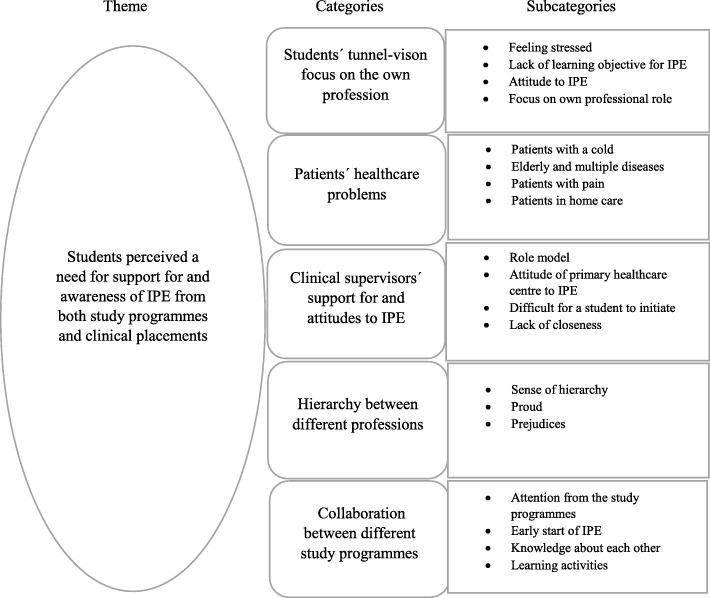
Students’ perceptions of IPE in primary healthcare: theme, categories and subcategories

A total of 26 students participated, 18 females and eight males. The mean age of the participants was 26.8 years (range, 25–44 years). Students represented medicine (n = eight), nursing (n = four), occupational therapy (n = nine), physiotherapy (n = five). See further Table 1. All students considered IPE in primary healthcare important. At the same time they found it difficult to collaborate in primary healthcare because the different professions did not work close to each other, and therefore it was difficult to get to know each other and begin collaboration. As an answer to our aim to describe how healthcare students perceived the conditions for IPE in primary healthcare, our results showed one theme that immersed all underlying categories: *Students perceived a need for support and awareness from both study programmes and clinical placements*. Five categories were found related to this main theme, representing the students’ perceptions of conditions needed for IPE in primary healthcare; *students’ tunnel-vision focus on their own profession, patients’ healthcare problems, clinical supervisor’s support and attitude to IPE, hierarchy between different professions* and *collaboration between different study programmes*. The main theme could be found in every category. Support and awareness from study programmes and clinical placements, could help students to change focus from their own profession to be more aware of other students’ professions. This support could facilitate for the students to get in touch with patients suitable for interprofessional learning. It could also break down hierarchy and provide role models for the students. This increased awareness and collaboration could facilitate for the students from different study programmes to meet and learn together more often. See further Fig. 1.

**Table 1:** Descriptive characteristics of the participating students

	All participants (*n* = 26)	N	OT	PT	M
Male	8	1	1	3	3
Female	18	3	8	2	5
Previous experiences in IPE	23	3	9	3	8

*N* nursing, *OT* occupational therapy, *PT* physiotherapy, *M* medicine, *IPE* interprofessional education

### Students perceived a need for support and awareness from both study programmes and clinical placements

This main theme that immersed the collected material, showed that study programmes and clinical placements were perceived by students as in need of support and increased awareness of IPE, otherwise IPE would not take place. According to the students, it felt strange that they did not see or collaborate with each other during their education but that they were expected to collaborate later when they had graduated. IPE was perceived important by all participants and they asked for more opportunities for IPE. They suggested some interprofessional learning activities, for example, case seminars and interprofessional discussions around patient cases. They thought that primary healthcare centres either facilitated or hindered IPE depending on what attitude the staff had towards IPE. The students also argued that something had to be done about the logistical problems. Most of the students interviewed did not meet any other students during their clinical placements. The students did not feel comfortable, without support from the programme or the clinical supervisors, to initiate IPE activities themselves in clinical environment.

I think there ought to be more – we have a lot of placements but I think there should be a bit more cooperation with the other programmes, that you should have the placements at the same time (occupational therapy student, group 1)

It must be brought into the education in some way, I mean actively, if it is to have any effect because otherwise nothing happens (medical student, group 3)

### Students’ tunnel-vision focus on their own profession

Students spent most of their time focused on learning their own profession and they had no time to spend on learning from other professions. They found it difficult to know what exactly their own professional role was at the clinical placements; hence they were busy attempting to find out how to act in their own profession. The students were so pre-occupied with learning the responsibilities of their own profession that this may have affected their ability to collaborate with students from other professions. The students believed that if they were expected to collaborate with other professions’ students, it would have made it easier for them to change focus from their own profession. During the interviews the students seemed unaware of their own attitude of not having time to learn from other professions. Some students felt stressed about having very short clinical placements of around a week, most often medical students, so they preferred to do things that only concerned their own profession.

I can imagine that it’s very much a matter of an attitude you have, even my own attitude, you know, that I come to the healthcare centre and I become rather narrow in my thinking because I say to myself, you know, what should I do to start thinking like a doctor (medical student, group 4)

You’re terribly locked, you know, you don’t think that you could do that too, or that you can follow someone else when you’re on a placement. You’re really geared to learning your own thing (occupational therapy student, group 1)

The students’ focus on their own profession was perceived as an obstacle to IPE.

### Patients’ healthcare problems

In primary healthcare, the students came across a wide range of patients and perceived that only a subset of the patients required collaboration with more than one profession. Many of the patients that the students had encounters with in primary healthcare had healthcare problems of low severity that did not seem urgent according to the students. Those patients who did not need collaboration came perhaps for just a cold, wanting antibiotics from the doctor, or they just wished to have their blood pressure measured by a nurse or to have a blood test in the laboratory. However, patients in primary healthcare with pain, elderly people with several diseases and complex problems who needed care in their homes, patients who were frequent visitors and patients with undiagnosed or not easily defined problems, were considered by the students as suitable for IPE. Those patients were perceived to need collaboration and it seemed to be more natural to involve other professions in the care of those patients.

But it’s a bit like this, some groups, you hear ‘I need a penicillin that works well’, and then maybe you don’t need to get involved, but if there’s someone who takes a lot more time or something like that, then you know there’s something to be found there (occupational therapy student, group 3)

Perhaps not those who come on one visit, but for the kind of people who come several times, to have more team conferences (medical student, group 4)

Some of the patients’ healthcare problems in primary healthcare were more suitable for IPE and others were not, according to the students.

### Clinical supervisor’s support and attitudes to IPE

Students did not often experience that the primary healthcare worked in teams or strove for collaboration because of several reasons. Different health professions had their own localities and each profession worked independently and collaborated mainly by referring the patient to the others when needed. However, the students perceived a need for role models. If their supervisor interacted with other professions the students also wanted to do so. The students perceived that the whole primary healthcare had to be a good example for the students by showing how to work in teams so that they were able to follow good examples of best practice. As a student they found it hard to introduce something new at a workplace. Nurses collaborated mainly with doctors while physiotherapists collaborated with occupational therapists, but those two different constellations rarely collaborated. One occupational student said it could happen that she never met a medical or a nursing student during her clinical placement.

In a place where they don’t work in teams, across professional boundaries, it can be hard as a student to get into the work and as a student to start working more in teams when you’re so fresh and you don’t have, you know (occupational therapy student, group 2)

I agree with that, actually, you get very much influenced by what your supervisor is like. If they focus only on their tasks and aren’t interested in collaborating with other professions, it ends up that you do the same yourself there (nursing student, group 4)

The students found it important that all the healthcare staff in primary healthcare were good role models for IPE.

### Hierarchy between different professions

The students had experiences of hierarchy between different professions in health care. In their opinion, hierarchy made people feel too proud of themselves and prevented them from seeking help from other professions. According to the students, prejudices existed among all professions. The students perceived that increased knowledge of other professions would help to prevent these prejudices and help to break down the hierarchy. In primary healthcare the professions worked in isolation. Therefore, the students suggested that students from different professions together with their supervisors would meet with the aim to increase knowledge about each other. When the different professions had gained an increased knowledge about each other, obstacles relating to hierarchy could be overcome. Further, students stated that hierarchy prevented the professions from communicating and reduced the opportunities to learn from each other. The students believed that hierarchy hindered them from seeing the other professions as resources.

Hierarchy creates so much status that’s unnecessary, you know, creates attitude in a way that makes cooperation difficult, I think (medical student, group 4)

According to the students, hierarchy between professions was an obstacle to collaboration and learning from each other.

### Collaboration between different study programmes

The students felt that there was lack of knowledge regarding the roles and responsibilities of other professions. The students would like to have had interprofessional learning activities together with other healthcare programmes. If they had the opportunity to learn more about each other from different programmes, it would have increased their willingness to ask for help from the other professions in future when they had graduated. They wanted to start early and carry on with interprofessional learning activities during their whole education. They thought that, in order to make them collaborate better with other professions, increased knowledge was required about each other. If they knew what knowledge and competence the other professions had, it would be easier for them to ask for help, refer to the other professions when needed and to collaborate. Otherwise, they rather preferred to ask their supervisors. Students perceived that their study programmes did not pay enough attention to IPE. Therefore, students needed to be reminded about IPE from their teachers in both verbal and written instructions, before their clinical placements.

That it’s more integrated somewhere in the education, that you get to learn what the others do, because I don’t know (nursing student, group 3)

The important thing is to bring it in more often and both early and late, I feel, because now you say it’s very late because we’re also in the last semester as physiotherapists. If, for example, you were to start perhaps together with doctors and have a course from the start, where you study theory together (physiotherapy student, group 1)

The students perceived a need to know more about each other from the beginning. They also wanted their teachers to remind them about IPE before their clinical placements.

## Discussion

The aim of this study was to investigate how students perceived the conditions for IPE in primary healthcare, and the main finding was that students perceived a need for support and awareness of IPE from both study programmes and clinical placements. This was essential since both study programmes and clinical environments could either hinder or enhance possibilities of IPE for students in primary healthcare. Students did not always encounter exemplary teamwork at their clinical placement. Our study showed, in accordance with a previous study by Thistlewaite [18], that if IPE was going to take place, it needed support and awareness from both study programmes and clinical placements.

Our most interesting finding was students’ tunnel-vision focus on their own profession. Since they were preoccupied with their own profession and wanted to only learn and do things related to their profession, this created an obstacle to learning with and from other professions. Fifty-five percent of medical students in the study by Morison, Boohan, Moutray and Jenkins [19] expressed that they did not want to waste time learning with other healthcare students but they did not explain the reason. Abu-Rish et al. [20] found that the most frequently reported barrier to interprofessional education implementation was scheduling, followed by difficulty in matching students of compatible levels. Our finding of students’ tunnel-vision focus on their own profession adds to these previous studies, since this finding has not been described previously.

Another interesting finding in our study was how the nature of the patients’ healthcare problems affected the students’ interest in IPE. Our findings differ considerably from studies made in hospital settings where patients were sicker or perceived as having health problems of a higher urgency. Several studies have mentioned that chronically ill patients need collaboration, which students in our study also suggested [21]. All students in our study agreed about interprofessional learning being crucial and important but complex and difficult for several reasons.

The students in our study called for interprofessional learning activities in concordance with the study by Tsakitzidis et al. [22]. According to the students in the study above, interprofessional learning should be included in undergraduate courses. The best timing for IPE whether early in the students’ education or later when they have formed their professional identities – is a question where there exists no consensus [23]. IPE should not be an “add-on” educational activity, it should be included in the curriculum because there could be problems regarding the time required for IPE [24]. Students from different study programmes usually have their clinical placements at different periods and most of the time the students do not meet students from other study programmes in the clinical learning environment. This logistical problem seems to be a challenge all over the world and difficult to solve [1, 25]. Chen, Delnat and Gardner [26] found that the lack of connection between education and clinical workplaces resulted in less opportunities for students to experience structured IPE in their clinical placement. Students in the present study appreciated meeting and having discussions with students from other professions, which rarely happened.

According to the students in the present study, clinical supervisors’ support and attitude towards IPE were important. The supervisors, through their support and attitude, could be either enablers or barriers to IPE since they acted as role models [27].

Students in our study experienced hierarchy among healthcare professionals, and from their perspective this hindered collaboration, communication, and shared knowledge; findings that were in concordance with the study by Abu-Rish et al. [20].

### Strengths and limitations

One strength of our study was that students from all four of the most common programmes in primary healthcare participated, which increased credibility. The research group came from different professions, two district nurses and one medical doctor (HS). There were also discussions between KB (an occupational therapist), and HS (a medical doctor), after every group interview. HS was responsible for the primary care component of the medicine study programme at Karolinska Institutet but had no direct contact as a teacher with the students in the present study. The interviews were not performed by the first author. It was not known whether the students felt equal to the other students during the interviews. It was likely easier to include the students who were interested in IPE than those with less interest. Another limitation was that three of the students had not had their clinical placement in primary healthcare so they spoke from their conceptions about how IPE in primary healthcare should be rather than actual experiences. Their contributions to the interviews were perceived as valuable so it was decided that their data would be included in the analysis. The present study was conducted in 2012. According to our knowledge, there have been no major changes in IPE in primary healthcare in Stockholm since this study was conducted. The pre-understanding of all the authors, who represent primary healthcare and teachers from the medical school with experience of working in primary healthcare, may have influenced in interpreting the collected data. Therefore, results and interpretations were continuously discussed between all authors until consensus was reached.

## Conclusions

Our results indicated that IPE was perceived as important but it required support and awareness from both study programmes and clinical placements. The study identified conditions that were required for IPE in primary healthcare. Our findings may be helpful for healthcare teachers and clinical supervisors to better understand how students perceive IPE in primary healthcare, thus facilitating the planning of IPE.

## Additional files

Additional file 1: Interview guide. (DOCX 12 kb)

## Abbreviations

IPE: Interprofessional education
