# Supplementary material for: Conditions for interprofessional education for students in primary healthcare: a qualitative study
Source: BMC Med Educ. 2018 Jun 4;18:122. doi: 10.1186/s12909-018-1245-8 (PMC5987484; doi:10.1186/s12909-018-1245-8)
Supplement: Supplementary file 1 — Interview guide. (DOCX 12 kb) [file 12909_2018_1245_MOESM1_ESM.docx]

**Interview guide**

1. What does IPE mean to you?
2. What possibilities do you see to learn with, from and about each other in primary healthcare?
3. What hindrances do you see to learn from each other in primary healthcare?
4. What can you learn from each other in primary healthcare?
5. How can you learn with and from each other in primary healthcare?
